# Efectividad y seguridad de las vacunas contra la Covid-19 en trabajadores de la salud en la Ciudad de Buenos Aires, Argentina

**DOI:** 10.31053/1853.0605.v81.n1.41155

**Published:** 2024-03-27

**Authors:** Vanina Pagotto, Facundo Seoane, Diego Arrigo, Victoria Ardiles, Valeria Asprea, Florencia De Florio, Analia Ferloni, Valeria Aliperti

**Affiliations:** 1 Departamento de Investigación del Hospital Italiano de Buenos Aires; 2 Hospital Italiano de Buenos Aires, Argentina; 3 Departamento de Cirugía, Hospital Italiano de Buenos Aires Argentina; 4 Departamento de Recursos Humanos. Hospital Italiano de Buenos Aires Argentina

**Keywords:** pruebas serológicas COVID-19, fuerza laboral en salud, vacunas contra la COVID-19, COVID-19 serological testing, health personnel, COVID-19 vaccines, testes sorológicos COVID-19, mão de obra em saúde, vacinas contra COVID-19

## Abstract

La vacunación COVID-19 ayuda a limitar la pandemia. En Argentina, se aprobaron tres vacunas y se priorizó a los trabajadores de la salud (TDS). El objetivo fue determinar la efectividad de las vacunas COVID-19, analizando el cambio en los niveles de Inmunoglobulina G y la incidencia de nuevos casos de COVID-19 hasta 12 meses después de la segunda dosis. Métodos: Cohorte prospectiva de TDS entre marzo de 2021 y 2022. Se utilizó la prueba COVIDAR IgG para medir los anticuerpos. Se empleó un modelo de efectos mixtos para comparar los niveles de inmunoglobulina G en diferentes puntos temporales, se utilizó Kaplan Meier para estimar los casos de COVID-19 incidentes. Resultados: se incluyeron 82 participantes. Los eventos adversos fueron frecuentes pero leves. Todos los participantes mostraron anticuerpos positivos a los 12 meses. Los niveles de anticuerpos mostraron un aumento un año después de la segunda dosis. Sinopharm tardó mucho tiempo en arrojar resultados positivos. Más de la mitad de las personas tuvieron una enfermedad leve de COVID-19. Conclusión: Las vacunas COVID-19 son seguras y efectivas.

## Abstract (en)

COVID-19 vaccination aids pandemic limitation. In Argentina, three vaccines are approved, and healthcare workers are priorized (HCWs). The aim was to determine the effectiveness of COVID-19 vaccines, analyzing change in Immunoglobulin G levels and the incidence of new COVID-19 cases up to 12 months after the second dose. Methods: Prospective cohort of HCWs between March 2021- 2022. COVIDAR IgG test was used to measure antibodies. A mixed-effects model was employed to compare the levels of immunoglobulin G at different time points, Kaplan Meier was used to estimate incident COVID-19 cases. Results: 82 participants were included Adverse events were frequent but mild. All participant showed positive antibody at 12 months. Antibodies levels showed an increase one year after 2nd dose. Sinopharm took a long time to yield positive results. More than half of the people had mild COVID-19 disease. Conclusion COVID-19 vaccines are safe and effective.

## Resumo

A vacinação contra a COVID-19 ajuda a limitar a pandemia. Na Argentina, foram aprovadas três vacinas e os trabalhadores da saúde (TDS) foram priorizados. O objetivo foi determinar a eficácia das vacinas COVID-19, analisando a mudança nos níveis de imunoglobulina G e a incidência de novos casos de COVID-19 até 12 meses após a segunda dose. Métodos: Coorte prospectiva de TDS entre março de 2021 e 2022. Foi utilizado o teste COVIDAR IgG para medir os anticorpos. Foi utilizado um modelo de efeitos mistos para comparar os níveis de imunoglobulina G em diferentes pontos temporais, e o Kaplan Meier foi utilizado para estimar os casos de COVID-19 incidentes. Resultados: foram incluídos 82 participantes. Os eventos adversos foram frequentes, mas leves. Todos os participantes apresentaram anticorpos positivos após 12 meses. Os níveis de anticorpos mostraram um aumento um ano após a segunda dose. A vacina Sinopharm demorou muito tempo para apresentar resultados positivos. Mais da metade das pessoas tiveram uma doença leve de COVID-19. Conclusão: As vacinas COVID-19 são seguras e eficazes.

**CONCEPTOS CLAVE**

**¿Qué se sabe sobre el tema?**

La gestión de emergencia de la epidemia de COVID-19 ha permitido la administración de vacunas sin haber completado los ensayos de fase 3. Es esencial conocer la efectividad a largo plazo en estudios del mundo real.

**¿Qué aporta este trabajo?**

Este estudio demuestra que las vacunas COVID-19 aprobadas en Argentina son seguras y tienen una respuesta de anticuerpos sostenida un año después de la vacunación. Los nuevos casos de COVID-19 después de la vacunación fueron frecuentes pero leves.

**Divulgación**

Este trabajo de investigación demostró la eficacia y seguridad en la vida real de las vacunas para COVID-19 más utilizadas en Argentina desde que se implementó la inmunización. Un año luego de la vacunación se observó la presencia de anticuerpos, lo que se considera una respuesta inmune sostenida. Los eventos adversos por las vacunas fueron frecuentes, sobre todo con la primera dosis pero leves. Más de la mitad tuvo COVID-19 luego de la vacunación, coincidiendo con la tercera ola epidemiológica de la variante Ómicron, pero ningún caso fue grave ni requirió internación.

## Introducción

La vacunación contra la enfermedad del coronavirus 19 (COVID-19) es una herramienta esencial para limitar los efectos de la pandemia. En diciembre de 2020, Argentina aprobó la vacuna Sputnik V, seguida de la vacuna recombinante ChAdOx1-S de AstraZeneca y la vacuna Sinopharm ^1^ . Aunque todas las vacunas demostraron eficacia en sus publicaciones ^2, 3^ , las mismas provienen de estudios de fase 3. En cambio, la efectividad se refiere a la protección proporcionada por la vacuna medida en estudios observacionales que incluyen a personas con condiciones médicas subyacentes que han estado recibiendo vacunas de diferentes proveedores de atención médica en condiciones del mundo real. Hasta ahora, son escasos los estudios epidemiológicos observacionales que revelen el comportamiento de los vacunados contra COVID-19 en Argentina y permitan la evaluación de su efectividad.

El Ministerio de Salud de Argentina diseñó, en diciembre de 2020, el "Plan Estratégico de vacunación contra COVID-19 en Argentina". La población priorizada en la campaña son los trabajadores de la salud (TDS), definidos como "cualquier persona que realice tareas y preste servicios en establecimientos de salud públicos o privados, independientemente de la relación contractual a la que estén sujetos" ^4^ . En Buenos Aires, Argentina, la campaña de los TDS comenzó el 29/12/2020.

Este estudio tuvo como objetivo determinar la efectividad de las vacunas COVID-19, incluyendo AstraZeneca/Covishield, Sputnik V y Sinopharm, mediante el análisis del cambio en los niveles de inmunoglobulina G contra los anticuerpos del SARS-CoV-2 y la incidencia de nuevos casos de COVID-19 hasta 12 meses después de la segunda dosis, dentro de la población de TDS.

## Materiales y Métodos

Se llevó a cabo una cohorte prospectiva de trabajadores de la salud (TDS) inmunizados con el esquema completo, es decir, dos dosis de vacunas COVID-19 entre marzo de 2021 y marzo de 2022.

Los participantes fueron invitados por correo electrónico institucional enviado por el comité de control de infecciones, y se evaluaron los criterios de

inclusión mediante una entrevista telefónica con el equipo de investigación.

Se recopilaron datos relacionados con la seguridad, eventos potencialmente atribuibles a la inmunización (ESAVI) después de recibir cualquier vacuna COVID-19, la historia de COVID-19 antes de la vacunación y la presencia de COVID-19 sintomático después de ella. El seguimiento se aseguró por contacto telefónico o por correo electrónico.

Se asumieron los títulos de anticuerpos de inmunoglobulina G (IgG) contra SARS-CoV-2 como parámetros de efectividad, medidos con la prueba "COVIDAR IgG", registrada en la Administración Nacional de Medicamentos, Alimentos y Tecnología Médica de Argentina (ANMAT). La prueba detecta anticuerpos en la sangre y suero que el sistema inmunológico produce para el nuevo coronavirus, específicamente contra dos antígenos virales: la proteína spike (S) y el dominio de unión al receptor (RBD) utilizando el test enzimoinmunoanálisis de adsorción. (ELISA), la prueba COVIDAR IgG detecta la presencia de IgG de forma cualitativa y semi-cuantitativa. Los valores se expresaron en Índice de Corte (COI), medidos como densidad óptica (OD). El COI se calcula dividiendo la OD por el valor de corte, siguiendo la ecuación:

COI = Control negativo*OD + 0,15

Los valores de COI <0,9 se consideran "NO REACTIVOS", los valores entre 0,9 y 1,1 se consideran "ZONA GRIS", y los valores superiores a 1,1 se consideran "REACTIVOS". El laboratorio de virología del Hospital Italiano de Buenos Aires procesó las muestras.

Las muestras para anticuerpos se realizaron en cinco medidas en el intervalo de tiempo según la vacunación COVID-19: primera medida (basal) antes de la primera dosis; segunda medida antes de la segunda dosis +- 2 días; tercera medida un mes +- 7 días después de la segunda dosis; cuarta medida después de seis meses +- 10 días de la segunda dosis y quinta medida después de 12 meses +- 15 días después de la segunda dosis.

El protocolo del estudio fue aprobado por el Comité de Ética de Investigación del Hospital Italiano de Buenos Aires el 18 de marzo de 2021, y registrado en ClinicalTrials.gov Identifier: NCT04843917.

### Análisis estadístico

Se empleó un modelo de efectos mixtos con términos de interacción para comparar los niveles de inmunoglobulina G en diferentes momentos y por tipo de vacuna COVID-19. Los términos de interacción se evaluaron mediante la prueba F y el valor p. Para abordar las comparaciones múltiples, se realizó la prueba de Tukey. La incidencia de casos de COVID-19 se estimó utilizando el método de Kaplan-Meier, y se compararon diferentes vacunas COVID-19 utilizando la prueba de log-rank. La significancia estadística se definió como valores de p inferiores a 0,05. El análisis se realizó utilizando el software R versión 4.2.3.

## Resultados

El estudio incluyó a 86 participantes que no habían recibido ninguna dosis de la vacuna COVID-19 hasta marzo de 2022. El análisis comprendió a 82 participantes, ya que cuatro fueron excluidos porque por razones personales no recibieron la segunda dosis. La mayoría de los participantes fueron inmunizados con la vacuna AstraZeneca/Covishield tanto para la primera como para la segunda dosis. Del mismo modo, quienes recibieron la vacuna Sinopharm, fue la misma en la primera y segunda dosis. De los 28 participantes que recibieron la vacuna Sputnik V, 11 recibieron una vacuna diferente como Moderna o Pfizer como segunda dosis. Las dosis de refuerzo fueron AstraZeneca, Moderna o Pfizer. Los eventos supuestamente atribuibles a la vacunación o inmunización (ESAVI) fueron en su mayoría leves y se resolvieron en un plazo de 72 horas. La Tabla 1 muestra las características basales de los participantes.

**Tabla 1:** Características de los participantes

	**N= 82**
**Hombres n (%)**	12 (14,6)
**Edad a la primera dosis media (DE)**	40,5 (10,4)
**Tipo de trabajo n (%)**	
Medicina	16 (19,5)
Enfermería	8 (9,7)
Técnico	11 (13,4)
Asistencia administrativa	36 (43,9)
Kinesiología	1 (1,2)
Instrumentación quirúrgica	6 (7,3)
Otros ^a^	4 (4,9)
**Tipo de jornada laboral n (%)**	
Presencial	44 (53,7)
Remota	8 (9,8)
Ambas	12 (14,6)

^a^arquitectura, terapia ocupacional, limpieza.

DE: desvío estándar; ESAVI: Eventos supuestamente atribuidos a la vacunación o inmunización.

Todos los participantes tuvieron anticuerpos IgG positivos 12 meses después de recibir la segunda dosis de cualquier vacuna COVID-19. La figura 1 muestra los cambios en los niveles de anticuerpos en cada medición. Después de ajustar por la primera medición (basal), ESAVI, el tipo de vacuna COVID-19, el género y la edad. Los niveles de anticuerpos globales (sin considerar el tipo de vacuna COVID-19, aumentaron en comparación con la segunda medición (figura 1A). Se observaron cambios comparables en los niveles de anticuerpos entre todas las vacunas COVID-19. Sin embargo, después de ajustar por la primera medición (basal), el género, la edad, ESAVI y COVID-19, se encontró que la vacuna Sinopharm exhibió niveles más bajos en las mediciones antes de la segunda dosis y a los 6 meses de la segunda dosis, en comparación con las otras vacunas (con una prueba F de interacción de 7,26 y un valor p de <0,001).

**Figura 1:** Cambios en los niveles de anticuerpos anti-SARS-Cov2 en cada medición. A) Global B) Para cada tipo de vacuna COVID-19.

Cuarenta y tres participantes contrajeron COVID-19, lo que resultó en una incidencia del 51,6% (IC del 95% 33-61) a los 8,6 meses. No hubo diferencia significativa entre las vacunas COVID-19 (p=0,720). Dado que todos los pacientes fueron reclutados en marzo-abril de 2021, esto fue consistente con la tercera ola epidemiológica de COVID-19, con el primer caso de la variante Ómicron detectado en Argentina el 5 de diciembre de 2021 ^5^ . Ninguno de los participantes desarrolló síntomas graves, requirió hospitalización o falleció.

La figura muestra los niveles de Inmunoglobulina G en el eje Y, medidos en índice de corte de densidad óptica (COI). Los puntos indican las medias y las barras indican los errores estándar. Las líneas punteadas muestran el límite de no detección. En la figura 1 A, las letras a, b y c indican que hay una diferencia estadísticamente significativa en comparación con la medición antes de la segunda dosis con un valor de p <0,001. En la figura 1 B, la letra c indica que hay una diferencia estadísticamente significativa entre la vacuna Sinopharm y la vacuna AstraZeneca/Covishield con un valor de p <0,001 y la letra d entre la vacuna Sinopharm y la vacuna SputnikV con un valor de p = 0,005.

## Discusión

Este estudio fue uno de los primeros en evaluar los efectos a largo plazo de las vacunas COVID-19 aprobadas en trabajadores de la salud en Argentina. Se encontró que las vacunas fueron seguras, incluso en un esquema heterólogo, con efectos secundarios frecuentes pero leves, similares a los descritos en otros estudios. ^6,7^ Aunque casi todos los participantes tenían anticuerpos IgG positivos contra SARS-CoV-2 seis meses después de la segunda dosis, aquellos que recibieron la vacuna de virus inactivado (Sinopharm) tenían niveles más bajos de anticuerpos comparados con las otras vacunas, lo que enfatiza la importancia de las dosis de refuerzo, especialmente para aquellos inmunizados con Sinopharm.

A los ocho meses de la primera dosis, se observó un 50% de casos nuevos de COVID-19, sin diferencia entre los tipos de vacunas COVID-19. La mayor incidencia de infecciones por COVID-19 en los participantes fue en marzo de 2021, después del verano de 2021-2022, con la variante Ómicron. Ninguno de los participantes experimentó enfermedad grave, hospitalización o muerte. Si bien los ensayos de fase III de las vacunas COVID-19 han demostrado menos infección en el grupo vacunado que en el grupo placebo ^2,3,8,9^, se reconoce ampliamente que estas vacunas previenen

principalmente la enfermedad grave y la muerte en lugar de la infección.

Es ampliamente conocido que las vacunas inactivadas por virus, como Sinopharm, pueden proporcionar menos protección que las vacunas vectoriales, como AstraZeneca/Covishield o Sputnik V ^10^ . Aunque los niveles de anticuerpos eran más bajos que los de las otras vacunas antes de la segunda dosis y a los 6 meses después, no hubo diferencias en los niveles o la tasa de enfermedad COVID-19 después de un año.

Este estudio tiene limitaciones y fortalezas. El tamaño de la muestra fue pequeño y solo consistió en personal de salud. Sin embargo, pudimos realizar un seguimiento de un año después de la segunda dosis.

Nuestros hallazgos demostraron una respuesta de anticuerpos sostenida para todas las vacunas COVID-19 aprobadas en Argentina, independientemente de la enfermedad COVID-19, los efectos secundarios o el tipo de vacuna. Más de la mitad de los participantes tuvieron una forma leve de enfermedad COVID-19 un año después de la vacunación, y los efectos secundarios fueron frecuentes pero leves. Además, la respuesta inmunológica se mantuvo un año después de la vacunación.
